# Towards improved health service quality in Tanzania: An approach to increase efficiency and effectiveness of routine supportive supervision

**DOI:** 10.1371/journal.pone.0202735

**Published:** 2018-09-07

**Authors:** Sabine Renggli, Iddy Mayumana, Dominick Mboya, Christopher Charles, Justin Maeda, Christopher Mshana, Flora Kessy, Fabrizio Tediosi, Constanze Pfeiffer, Alexander Schulze, Ann Aerts, Christian Lengeler

**Affiliations:** 1 Swiss Tropical and Public Health Institute, Basel, Switzerland; 2 University of Basel, Basel, Switzerland; 3 Ifakara Health Institute, Dar es Salaam/Ifakara, United Republic of Tanzania; 4 Africa Centres for Disease Control and Prevention (Africa CDC), Addis Ababa, Ethiopia; 5 Ministry of Health and Social Welfare, Dar es Salaam, Tanzania; 6 Swiss Agency for Development and Cooperation, Berne, Switzerland; 7 Novartis Foundation, Basel, Switzerland; Tulane University School of Public Health and Tropical Medicine, UNITED STATES

## Abstract

Effective supportive supervision of healthcare services is crucial for improving and maintaining quality of care. However, this process can be challenging in an environment with chronic shortage of qualified human resources, overburdened healthcare providers, multiple roles of district managers, weak supply chains, high donor fragmentation and inefficient allocation of limited financial resources. Operating in this environment, we systematically evaluated an approach developed in Tanzania to strengthen the implementation of routine supportive supervision of primary healthcare providers. The approach included a systematic quality assessment at health facilities using an electronic tool and subsequent result dissemination at council level. Mixed methods were used to compare the new supportive supervision approach with routine supportive supervision. Qualitative data was collected through in-depth interviews in three councils. Observational data and informal communication as well as secondary data complemented the data set. Additionally, an economic costing analysis was carried out in the same councils. Compared to routine supportive supervision, the new approach increased healthcare providers’ knowledge and skills, as well as quality of data collected and acceptance of supportive supervision amongst stakeholders involved. It also ensured better availability of evidence for follow-up actions, including budgeting and planning, and higher stakeholder motivation and ownership of subsequent quality improvement measures. The new approach reduced time and cost spent during supportive supervision. This increased feasibility of supportive supervision and hence the likelihood of its implementation. Thus, the results presented together with previous findings suggested that if used as the standard approach for routine supportive supervision the new approach offers a suitable option to make supportive supervision more efficient and effective and therewith more sustainable. Moreover, the new approach also provides informed guidance to overcome several problems of supportive supervision and healthcare quality assessments in low- and middle income countries.

## Introduction

Improving health service quality is a prerequisite for moving towards Universal Health Coverage and therewith crucial for achieving the health-related Sustainable Development Goal 3 [1, 2]. Various quality improvement initiatives have been implemented in resource constrained environments [3], including supportive supervision. Supportive supervision can be understood as on-site supervision or mentorship usually provided by health authorities under a supportive or facilitated model, with immediate feedback to the healthcare provider to assist in improving the performance [4, 5]. Supportive supervision was shown to promote quality improvements for structural and process elements in a number of low resource settings [5–14]. However, systematic reviews on this topic found mixed evidence on its effect on quality of care [5, 15]. This suggests that effective supportive supervision of healthcare services strongly depends on the way it is conducted, as well as on contextual factors [5, 9, 16]. It is seen as particularly challenging in an environment with a chronic shortage of qualified human resources, overburdened healthcare providers, multiple roles of district managers, weak supply chains, high donor fragmentation and inefficient allocation of limited financial resources [5, 17]. These factors also describe well the challenges faced by the Tanzanian health system [18, 19].

In Tanzania, Regional Health Management Teams (RHMTs) have the responsibility to supervise Council Health Management Teams (CHMTs) and ensure implementation of routine CHMT supportive supervision [20, 21]. CHMTs are supposed to conduct supportive supervision in all hospitals, health centres and dispensaries within their council on a quarterly basis [20]. They are also in charge of developing the annual Comprehensive Council Health Plan (CCHP), which includes operational plans and budgets and is based on routinely collected health information and supportive supervision findings [22]. According to the concept of Integrated Management Cascade (IMC), the health centres should carry out supportive supervision of dispensaries within their catchment area [20]. At facility level the Health Facility Governing Committees (HFGCs), composed of community representatives, oversee the facility operations [22, 23]. Likewise, at council level the Council Health Service Board (CHSB), consisting of community and private health sector representatives, is the governance body responsible for CHMT oversight and CCHP approval before its submission to the full council assembly [22, 23].

Routine CHMT supportive supervision has often been reported as infrequent, inefficient and ineffective in tackling performance gaps [10, 20, 24–28]. Although national supportive supervision guidelines exist, they are not followed in practice [20]. Also, councils have been using a general supportive supervision checklist to develop their own list, which makes comparison between councils impossible [20]. Routine CHMT supportive supervision concentrates on quantity (reviewing record books) with insufficient focus on quality elements (delivery processes) [20, 25, 28–30]. It is often more of an inspection, whereas the supportive element is hardly practiced [25, 27, 31]. Supportive supervision was also reported as fragmented, incomplete and inconsistent with no or solely negative feedback [20, 24, 25, 28, 29, 31–34]. CHMTs struggle to systematically follow-up and report back about issues identified during supportive supervision [32, 33]. Additionally, there is a lack of accountability of CHMTs to RHMTs and supervision of CHMTs by RHMTs is weak [20, 21].

Overall, poor routine CHMT supportive supervision has been reported to slow down progress in quality improvement, negatively affecting job satisfaction, staff presence, performance, motivation and retention as well as impairing other quality improvement interventions [11, 24–26, 29, 31, 33, 35, 36]. There are several strategic documents in Tanzania emphasising the need for enhanced supportive supervision in order to improve quality of healthcare services [20, 21, 27, 28].

**Fig 1:**
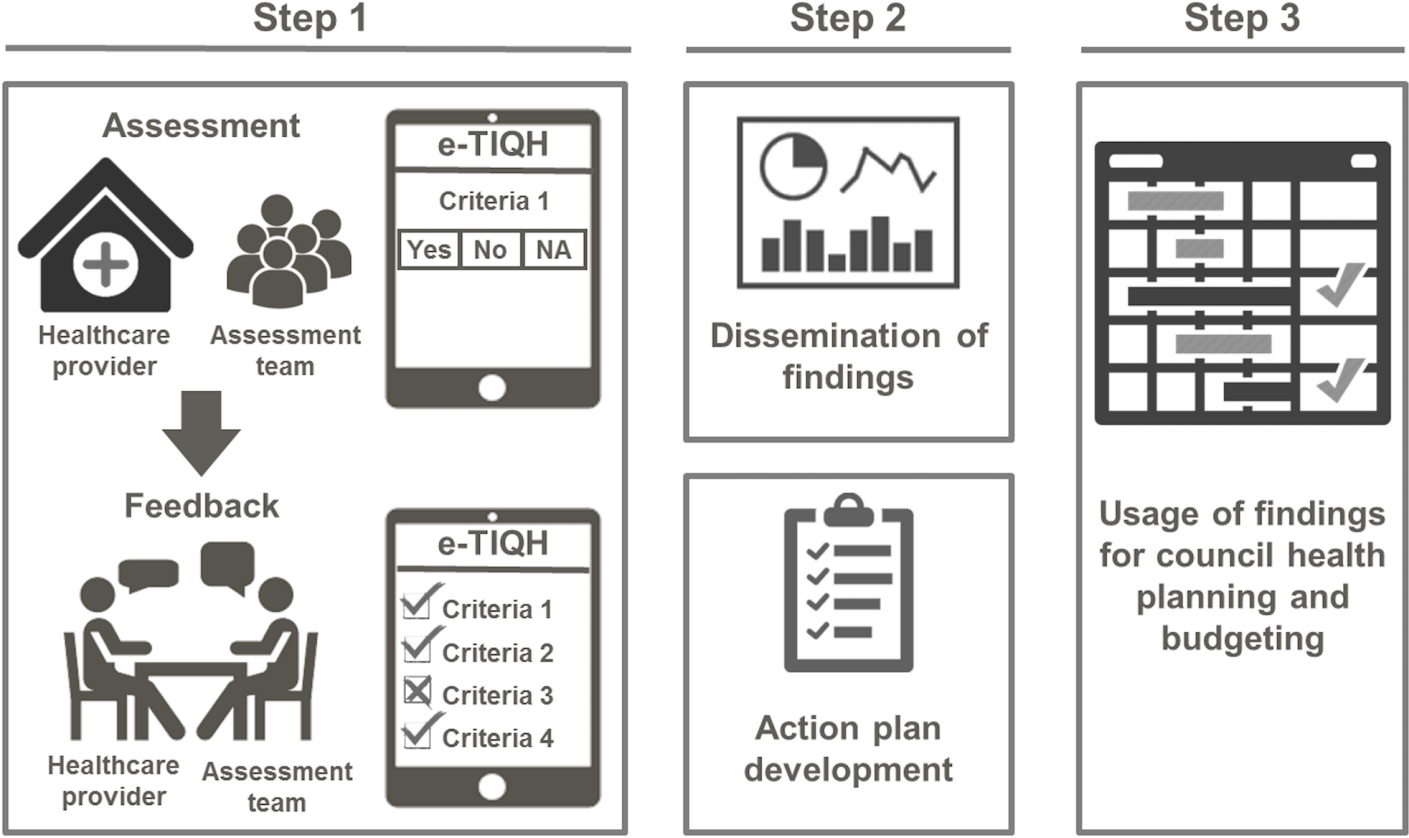
Chart of the three-stage process of the e-TIQH supportive supervision approach [37].

To inform council implementation of supportive supervision, we systematically evaluated a three-stage approach developed in Tanzania as part of the “Initiative to Strengthen Affordability and Quality of Healthcare (ISAQH)”. The aim of the approach was to improve quality of primary healthcare through strengthening the implementation of routine CHMT supportive supervision of healthcare providers. In a first step regular systematic assessments of quality of primary care were carried out in all health facilities within a given council, using the “electronic Tool to Improve Quality of Healthcare”–in short e-TIQH (Fig 1).

CHMT core and co-opted members formed the core of the assessment team, but to increase objectivity and reduce bias community representatives and healthcare providers from the public and private sector were involved as well. Assessment supervision was done by ISAQH staff. The assessment methods included checklists, structured interviews and direct clinical observations, focusing on processes and structural adequacy of healthcare [38]. Using a clearly defined and concise set of indicators, six quality dimensions were assessed: [1] Physical environment and equipment, [2] Job expectations, [3] Professional knowledge, skills and ethics, [4] Management and administration, [5] Staff motivation, and [6] Client satisfaction. Points were given for each indicator met within a dimension, and percentage scores (of total possible points) were calculated per quality dimension. Importantly, the assessment concluded with an immediate constructive feedback to the healthcare providers, joint discussions about how to address the identified quality gaps, and the provision of a short written feedback summary form for the health facility. In a second step, a dissemination meeting was held at council level with all relevant stakeholders to discuss the findings and develop action plans. This provided important inputs for the third step, the annual CCHP development process of the CHMT.

**Fig 2:**
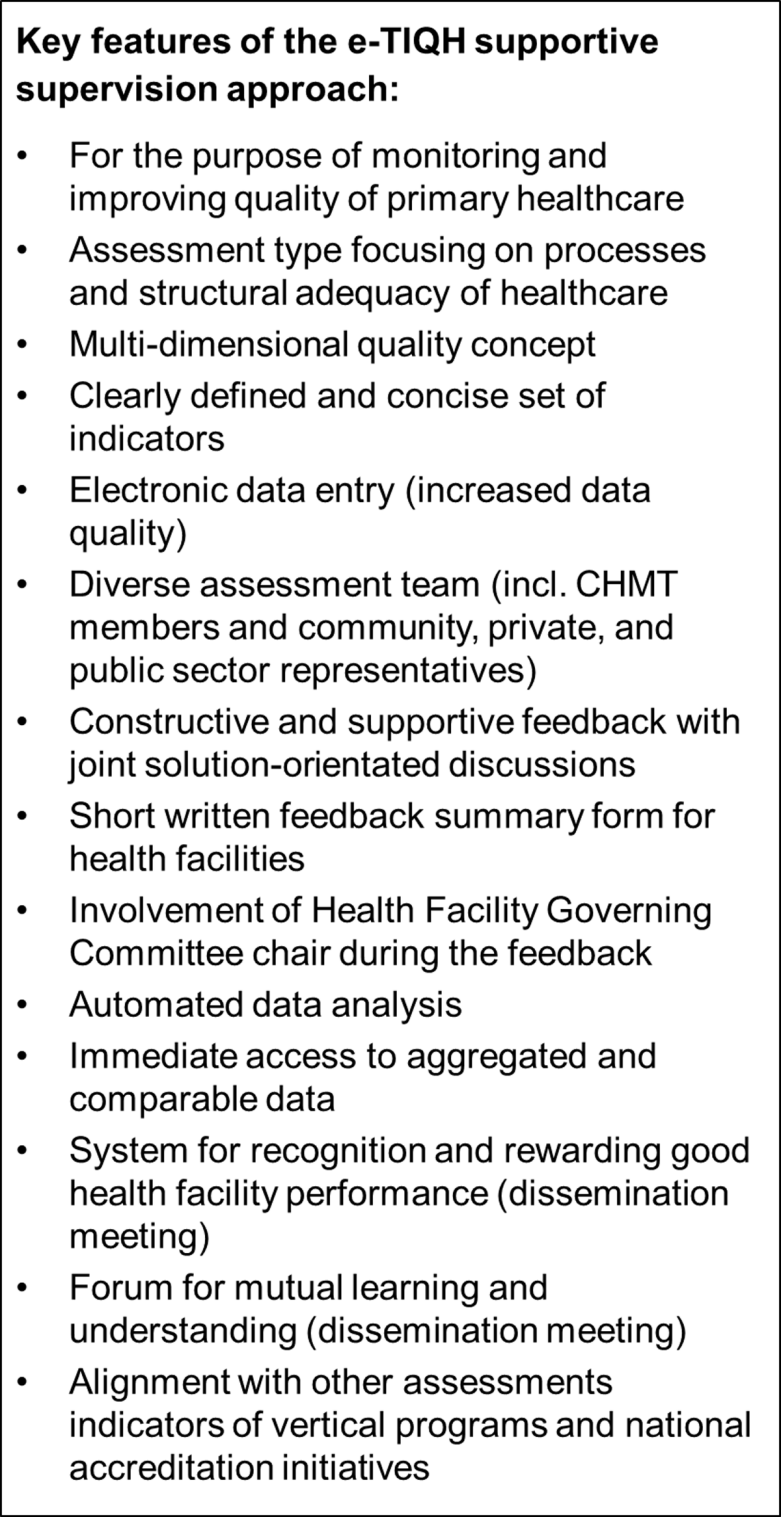
Key features of the e-TIQH supportive supervision approach [37–39].

The supportive supervision approach and in particular the e-TIQH assessment tool as an integral part have already been described in detail by Mboya et al. [37]. Fig 2 summarizes its key features.

The appropriateness of the e-TIQH assessment tool to measure and monitor quality of primary healthcare was assessed using a range of different quantitative and qualitative methods and reported elsewhere [38]. Additionally, it has been shown in another publication on the same topic [39] that the e-TQIH supportive supervision approach could improve and maintain crucial primary healthcare standards and therewith contribute to increased quality of care. This paper now aims to assess the suitability of the e-TIQH supportive supervision approach to improve the currently implemented routine CHMT supportive supervision approach. Therefore, a comparison of the implementation of the two approaches will be done.

## Materials and methods

### Study setting

The mixed method approach, which was used to compare e-TIQH and routine CHMT supportive supervision, consisted of a qualitative study and a costing study conducted in the first quarter of 2016. Activities implemented during each of the two supportive supervision approaches were reported by the study participants of either study and thus summarized in the result section.

**Fig 3:**
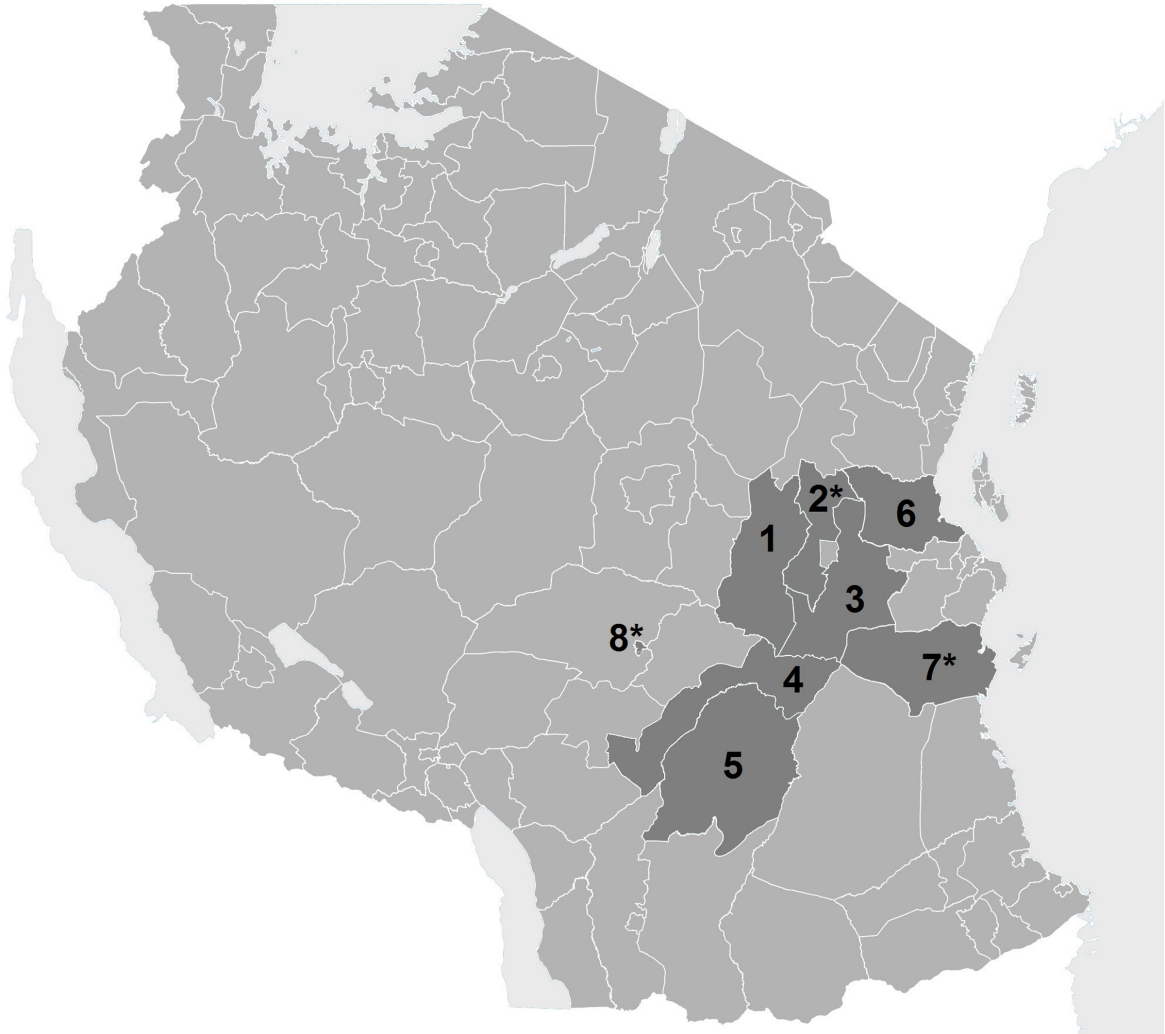
Map of Tanzania with councils where the e-TIQH supportive supervision approach was implemented (status 2008). Morogoro Region: (1) Kilosa DC (later split into Kilosa DC and Gairo DC), (2) Mvomero DC, (3) Morogoro DC, (4) Kilombero DC, (5) Ulanga DC; Pwani Region: (6) Bagamoyo DC, (7) Rufiji DC; Iringa Region: (8) Iringa MC. Asterisks mark the three study councils.

Fig 3 presents a map of Tanzania with district and municipal councils (DC and MC) where the e-TIQH supportive supervision approach (with all its features as describe in the Fig 2) was implemented in parallel to the routine CHMT supportive supervision approach. For the purpose of the studies described here, three out of these eight intervention councils were purposefully selected (see asterisks in Fig 3).

These three councils were originally chosen for the purpose of another study described elsewhere [39] due to their most pronounced yearly increases in overall quality (as measured by the e-TIQH assessments) compared to the other councils. However, because sampling coincidentally resulted in the selection of three councils, which were very different in terms of their characteristics (Table 1) and thus representative for wide range of contexts within Tanzania, we decided that the three councils also perfectly suited the purpose of this study.

**Table 1:** Description of councils selected for the study.

Characteristics	Rufiji DC	Mvomero DC	Iringa MC
Region	Pwani	Morogoro	Iringa
Classification	rural	rural	urban
Population size [40]	217'274	312'109	151'345
Area (km^2^)^1^	13'339	7'325	162
Road (km) [41]^2^	467	289	178
Accessibility	Several hard-to-reach areas, including the Rufiji river delta	Some hard-to-reach areas	No hard-to-reach areas
Number of operating health facilities (hospital/ health centres/ dispensaries) [42]^3^	78 (2/6/70)	69 (3/8/58)	33 (3/4/26)
Existence of pay for performance (P4P) schemes [29]^4^	Pilot council for donor funded P4P scheme since 2011	Partially implemented locally funded P4P scheme between 2009 and 2011	No P4P experience
National star rating system in place since 2016 [18]^5^	Yes	No	No

^1^Source: Comprehensive Council Health Plans of participating councils collected by SR and IM

^2^gravel, tarmac, earth

^3^status October 2016

^4^Result-based financing scheme whereby financial incentives, which are tied to the achievement of service coverage and/or quality improvements, are provided to the healthcare provider

^5^A performance-based certification system implemented by Ministry of Health and Social Welfare under the Big Results Now initiative

### Qualitative data

To identify advantages and disadvantages of the routine CHMT and e-TIQH supportive supervision, a qualitative methodological approach was taken. The main part of the qualitative data consisted of in-depth interviews. Observational data and informal communication recorded in a field notebook together with secondary data collected during the field work complemented the data set. Secondary data included copies of health facility guest books as well as CCHPs, quarterly combined Technical and Financial Performance Implementation Reports (TFPIRs), council routine supportive supervision checklists and reports. In total, 24 in depth-interviews were conducted in the three study councils (Fig 3). Sampling of interview partners was done purposefully as described elsewhere [39]. In order to be considered as a respondent, the respondents had to be in their respective position at least for part of the time period in which the e-TIQH approach had been implemented. If no such respondent was available, a respondent with comparable experience, based on the interviewers’ judgement, was selected. The characteristics of the selected respondents are summarized in Table 2. For confidentiality reasons, no further information about the respondents could be given.

**Table 2:** Number of in-depth interviews done in the three study councils (Mvomero DC/ Rufiji DC/ Iringa MC).

Position	Administrative level	Sector	
		Public	Non-public
CHMT (co-opted) member	Council	2/2/2	
CHSB member	Council		2/2/2
Health centre in-charge	Health centre	1/1/0	
Quality improvement person	Health centre	1/1/0	
Dispensary in-charge	Dispensary	2/2/2	0/0/2
**Total**		16	8

Interviews were conducted in Swahili and led by a Swahili speaking female Swiss (SR), backed up by a male native Tanzanian of middle age (IM). Both were familiar with the team facilitating the implementation of the e-TIQH supportive supervision approach, but were never part of it. In order to identify advantages and disadvantages of either approach, respondents were asked to describe routine CHMT supportive supervision and then compare it with e-TIQH supportive supervision. Additionally, we probed for potential activities conducted during preparation, implementation, reporting and dissemination as well as for data usage upon completion of supervision visits. All interviews were tape-recorded and transcribed by two native Tanzanian research assistants but not translated into English. The Swahili transcripts were managed and coded using MAXQDA software. Data were analysed according to Gale’s framework analysis [43]. Codes were primarily developed inductively. After repeated reading of transcripts and initial coding, emerging themes were structured to obtain a coding framework. The theme ‘Quality of data collected’ was split deductively into the categories proposed by the WHO guide to improve data quality (accuracy, reliability, completeness, legibility, timeliness, accessibility, meaningfulness and security) [44]. Within a category codes were assigned to the supportive supervision type (routine CHMT or e-TIQH) and the activity (Preparation, implementation, reporting, dissemination) they described. Statements of a given category and activity were then compared between the supportive supervision types to conclude a perceived improvement or decline when switching from routine CHMT to e-TIQH supportive supervision. Findings were also compared for similarities and differences within and between respondents attributes such as gender, age, position as well as their working environment (council, level and ownership of health facility). Finally, citations quotes used in this publication were translated by SR into English and proofread by IM.

### Cost assessment

In order to complement and strengthen the qualitative data collected, we compared the cost of routine CHMT and e-TIQH supportive supervision by calculating quarterly recurrent council level cost for each of them. Therefore, an economic costing was carried out, identifying the value of all resources required to conduct supportive supervision. To do so an ingredient approach was employed, whereby quantities of each resource were identified, measured, and valued with the appropriate unit cost [45]. Costs were classified by type of resource (personnel, per diem/allowance, transport, other expenses) and activity. To identify the activities done, time spent and resources used, three to four CHMT members in each of the three study councils were interviewed. In order for their statement to be valid, they had to be participating in the corresponding activity of both approaches. Council routine supportive supervision checklists and reports were collected as a reference. For the e-TIQH approach, time estimates were cross verified with observations done by ISAQH staff during implementation.

Personnel cost was defined as the cost of staff time and estimated based on their salaries according to national salary scales and time spent [46]. Other unit costs were derived from information given by respondents, CCHPs, quarterly combined TFPIRs as well as ISAQH documents, other official documents collected and personal communication. Market prices were taken to value supplies (S1 Table). Cost spanning multiple quarters were equally divided over the relevant time period. One time start-up cost to develop the e-TIQH online platform of 113’680 USD was not included. The estimation of regional and national level cost was outside the scope of this study. All costs were calculated in Tanzanian shillings and converted to USD using the annual average exchange rate for 2016 (2’188TSh = 1 USD) [47].

The overall activity of doing supervision was broken down into activities prior to (preparation), during (implementation) and after (reporting, dissemination) supportive supervision visits (S2 Table). To estimate the required number of assessment days, time needed at each health facility level (dispensary, health centre, hospital) was calculated. Travel time between health facilities and their typical distribution in a council were also taken into account. Assessment days were integrated into the cost calculations as a full working day (eight hours), even if adding up the time spent at health facilities was less. Due to the fact that at council level no activity equivalent to the result dissemination meeting could be identified, this cost was calculated separately. The same was done for start-up costs to introduce the e-TIQH approach in a council. Time estimations for these two activities were taken from the ISAQH records. All costs were calculated for an average rural and urban council (Table 3).

**Table 3:** Relevant characteristics of an average rural and urban council in Tanzania.

	Rural (N = 136) [42]^1^	Urban (N = 40) [42]^2^
Total number of health facilities [42]	40	30
*Hospital*	1	2
*Health centre*	4	5
*Dispensary*	35	23
Distance to be covered (km)^3^	3'500	1'400

^1^Includes all District Councils

^2^Includes all Town, Municipal and City council, except the three Town Councils of Dar es Salaam

^3^Estimation based on the fuel consumption during the implementation of the e-TIQH supportive supervision approach

For the e-TIQH supportive supervision approach two options were calculated: recommended and reduced assessor option. The recommended option consisted of two more assessors (two teams of six) than the routine CHMT approach (two teams of five), where the two additional assessors were non-CHMT members (e.g. CHSB members, private sector representatives). In order to facilitate the comparison to routine supportive supervision, the reduced assessor option involved the same number of assessors as the routine conventional approach (two teams of five). Reducing the assessment team by one assessor, would not affect the total time spent at a health facility due to the fact that e-TIQH quality dimension 1 was assessed as a team and subsequently quality dimensions 2 to 6 were evaluated concurrently by one assessor each.

### Ethical considerations

Permission to publish the findings was obtained from the National Institute for Medical Research (NIMR) in Tanzania. Ethical clearance was granted by the same institution (original: NIMR/HQ/R.8a/Vol.IX/1839, extension: NIMR/HQ/R.8c/Vol.II/521), the Institutional Review Board of the Ifakara Health Institute (IHI/IRB/No:37–2014) and the Ethic Commission of Northeast and Central Switzerland (EKNZ 2014–347). For the in-depth interviews written informed consent and for the costing oral informed consent was obtained from all respondents.

## Results

**Fig 4:**
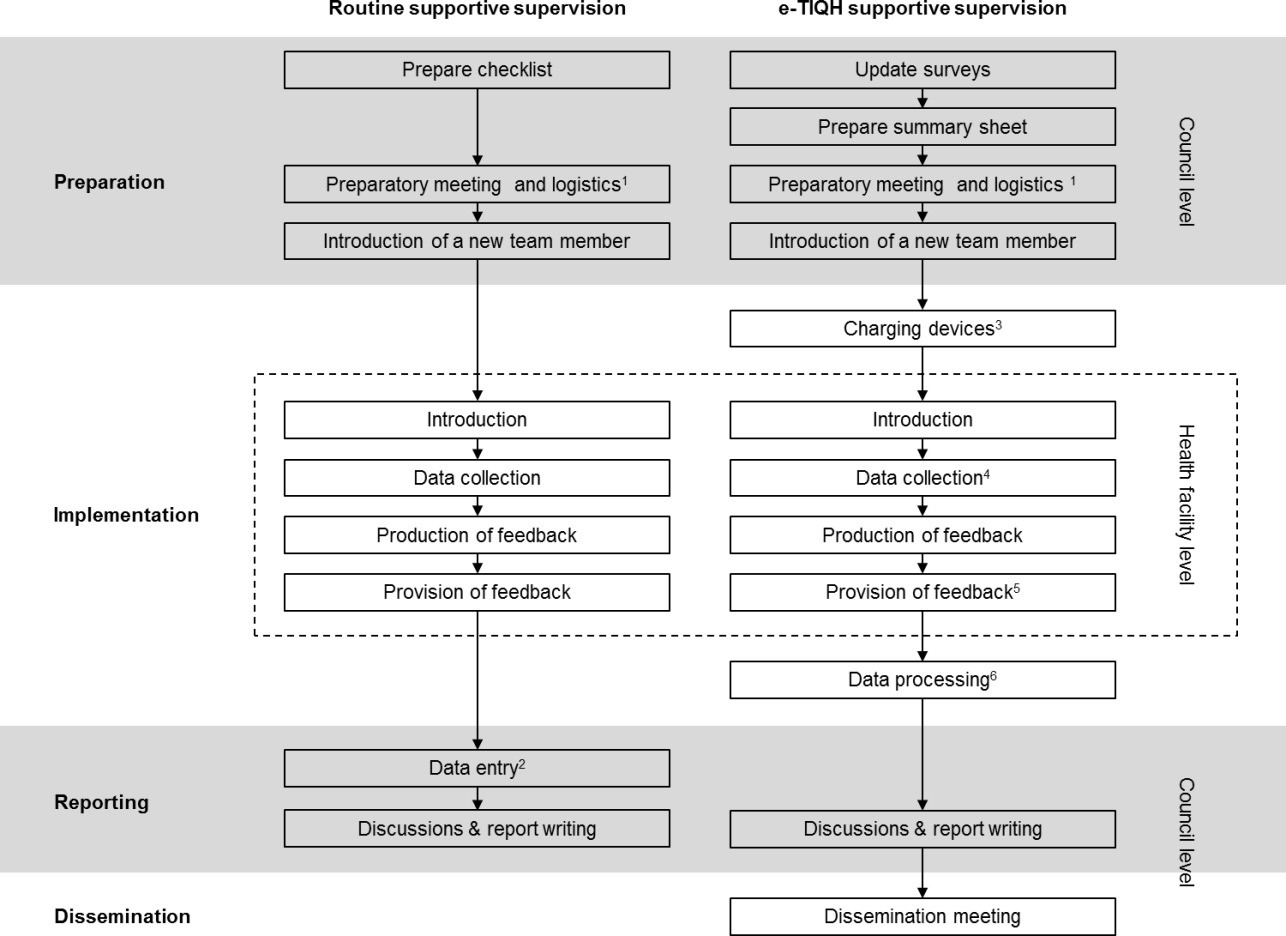
Activities conducted during routine CHMT and e-TIQH supportive supervision. ^1^The preparatory meeting included setting up the teams and their routes; logistics included informing health facilities and request transport and per diems; ^2^Data entry after routine CHMT supportive supervision was hardly ever done; ^3^Charging devices was reported to take seven minutes for six tablets per team and day; ^4^Quality dimension 1 was evaluated as a team and subsequently quality dimensions 2 to 6 were assessed concurrently by one assessor each; ^5^Provision of feedback included the completion of five page feedback summary form; ^6^Estimated time for data processing (quality check and uploading survey forms) was one and a half hours per team and day.

In the qualitative and the costing study, respondents were asked to describe the activities of the two supportive supervision approaches. Fig 4 summarizes activities reported to be conducted during routine CHMT and e-TIQH supportive supervision. Preparation, reporting and dissemination were done at council level and actual implementation at health facility level. An important finding was that data collected during routine CHMT supportive supervision was hardly entered upon return due to shortage of human resources, time limitation and competing priorities.

### Qualitative data

**Fig 5:**
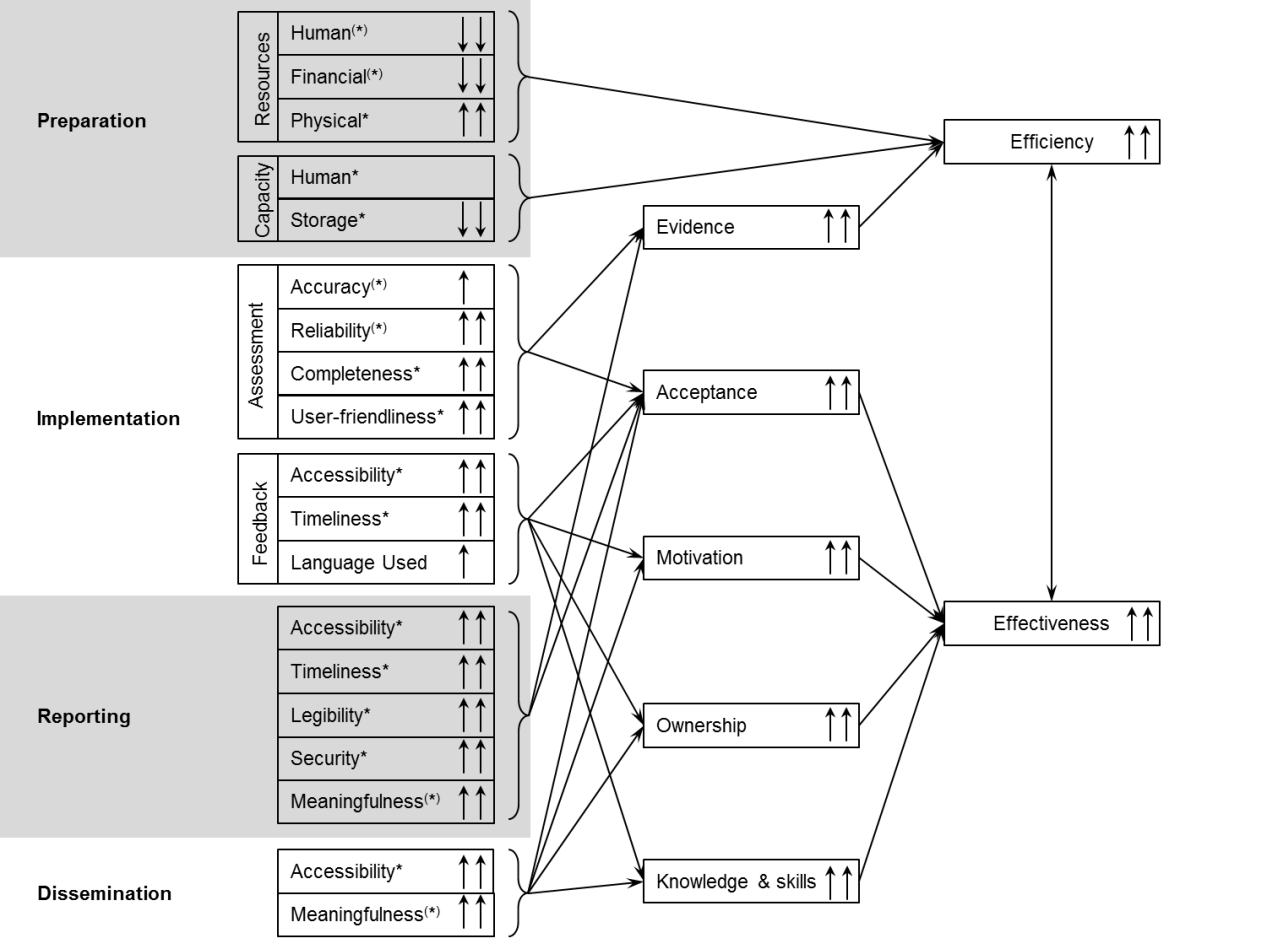
Comparison of routine CHMT and e-TIQH supportive supervision. Upwards arrows show a perceived improvement and downwards arrows a perceived decline when switching from routine CHMT to e-TIQH supportive supervision. Perceived change based on the qualitative data (statements given frequently and/or across administrative levels and sectors) is given by single (likely change) and double (clear change) arrows. Asterisks indicate that the particular change could primarily* or partially^(^*^)^ be attributed to the usage of an electronic tool per se. For items without an asterisk or an asterisk in brackets ^(^*^)^, the overall e-TIQH supportive supervision approach was relevant as well. For physical resources it was assumed that tablets need be bought.

A total of 23 out of 24 respondents directly experienced routine CHMT (21 of 23), e-TIQH (22/23) or both (20/23) supportive supervision as an assessor or as the person being assessed. The following analysis will be restricted to these 23 people, since only they could state advantages and disadvantages of either approach. In order to link the qualitative data with the cost assessment, the subsequent section was structured according to the activities reported to be conducted during supportive supervision (Fig 4) and findings are summarized in Fig 5.

#### Preparation–resources and capacity needed for implementation

For the routine CHMT supportive supervision infrequent implementation was reported and observed several times (13/23, observational data). Main reasons for this were lack of time (human resources) due to competing tasks (11/23, observational data) as well as insufficient and untimely financial resources because of cumbersome administration processes at council level and delayed or inadequate disbursement of money from the central government (10/23, observational data). This was illustrated by a CHMT member as follows:

“Doing it [supportive supervision] has its challenges. We have… competing tasks. You do a schedule [which shows] that the whole week we dedicate to go to the health facilities for supportive supervision but in between some CHMT members are called for a certain seminar… Or we get visitors from the ministry, different organisations, NGOs that we work with. Thus, some of us need to go there, join them to do some work. Hence, you come to realize this week is lost and… maybe you managed to just visit one facility… these have been our big challenges…” (CHMT member, Iringa MC)

In contrast, for the e-TIQH supportive supervision approach financial resources were readily accessible through project funds. Also, several respondents stated that e-TIQH supportive supervision required less time at the health facility (10/23) and for reporting (4/23), supporting findings of the cost assessment below. This was clearly attributed to the electronic nature of the assessment and the more concise list of indicators compared to routine CHMT supportive supervision.

Additionally, some respondents (3/23) elaborated from a provider perspective how the e-TIQH assessment not only reduced time required, but also the time burden of supportive supervision as explained by a CHMT member:

“The e-TIQH assessment often doesn’t involve all the staff… Sometimes it’s not necessary [to directly work with] the facility in-charge… an experienced person can show us all the places… Therefore, the rest of the work continues as normal. Also, because you use the tablets it doesn’t take a lot of time… But for the one [supportive supervision] of the CHMT… it means the service stands still (…) But for e-TIQH you go and people go on with the work. (…) Then, to interview someone it doesn’t take a lot of time because once s/he answered, you just enter it [into the tablet].” (CHMT member, Rufiji DC)

If the e-TIQH approach was to be implemented by the CHMT alone, the main concerns regarding resources were the affordability of tablets (8/23), of the dissemination meeting (4/23) and of the platform running cost (observational data) as well as the availability and affordability of non-CHMT assessors (2/23, observational data). However, as presented in findings of the cost assessment below, these concerns could not be confirmed by the costing data apart from the cost of the dissemination meeting.

In terms of human capacity, for both approaches respondents emphasized the importance of well-trained assessors with the required contextual knowledge and professional skills to conduct the assessment (7/23), but also the organisational skills to ensure smooth implementation of the approach (11/23, observational data). Both issues were seen to be lacking to some extent during routine CHMT supervision, but not during e-TIQH supportive supervision. In addition, for the e-TIQH approach basic IT skills for managing the electronic devices were perceived as necessary (5/23), but less analytical skills were reported to be required due to automated data analysis (4/23). However, interviews and observations also revealed some inability to fully use the results generated at aggregated level by those in a position to do so (3/23).

#### Implementation

Respondents said that a main advantage of the e-TIQH assessment tool was its design with the emphasis on key issues of primary healthcare (8/23), the wider range of topics assessed (e.g. staff motivation, patient satisfaction) (18/23) and the assessment type, which focused on adequacy and processes of care (clinical observations) (16/23) [38]. A facility in-charge summarized this as follows:

“There are a lot of supervisions being done, but they [e-TIQH assessors] want to observe [the service delivered]. It’s not like we sit and you ask [if] a certain thing [process] is being done. He [the e-TIQH assessor] wants to see if you are really doing it. If you say the guidelines are there, ‘Where are they?’ It’s not [possible] to say they are there, but they aren’t. He looks exactly where they are. (…) However, when they [CHMT] come, they look how you fill [the register book]. Thus, they don’t look how you did the diagnosis of this patient… but they look how you filled [the record books]. (…) But he [e-TIQH assessors] wanted to see how the clients are being attended, and how at the same time the data was entered [into the record books]. (…) But they [CHMT] do supervision by simply asking questions independent of whether or not there is a client.” (Facility in-charge, Iringa MC)

Moreover, it was seen as less biased (12/23) due to a more diverse and skilled assessment team (8/12) and the usage of an electronic tool (8/12). This was illustrated by a CHSB member:

“The nice thing about the [e-TIQH] approach was [that] it used a mixed group of assessors, it took people form the private [sector] to go and do supervision even at public [facilities]. So, this takes away biases.” (CHSB member, Rufiji DC)

As a result of improved assessment design and reduced bias, most people perceived the overall e-TIQH assessment as more accurate than routine CHMT supportive supervision (15/23). Observations and interviews with CHMT members revealed that checklists of routine CHMT supportive supervision were often more extensive and covered more types of medical services (8/23, observational data). However, mainly health facility record books and availability of care were investigated, whereas adequacy was only assessed to some extent and processes hardly ever (11/23; observational data). Thus, the increased perceived accuracy of the e-TIQH assessment tool was not necessarily due to a higher number of indicators. However, the perception of increased accuracy led to higher acceptance of the assessments and their results amongst stakeholders involved (18/23).

Reliability and completeness of routine CHMT supportive supervision data was reported to be strongly affected by inconsistent data collection (14/23, observational data). This was seen to be due to insufficient human resources, which resulted in a constantly changing composition of the assessment team (4/14) and lack of time to go through an extensive supervision checklist (13/14).

In contrast, owing to a clearly defined, fixed and more concise set of indicators as well as the electronic nature of the tool, e-TIQH assessment reliability was perceived higher and data completeness was not an issue (9/23, observational data). The electronic tool was also seen as more user-friendly compared to the paper-based assessment (9/23). In terms of feedback given, at health facilities interviewees stated that it was more adequate and constructive (20/23). This was said to be due to the more supportive attitude and language of the assessors (15/20) as well as the immediate availability of initial analyses thanks to the electronic format of the assessment (timeliness and accessibility of data) (11/20).

Thus, according to the respondents, acceptance of the feedback (16/23), ownership of the actions to be taken at facility level (12/23) and staff motivation (10/23) increased. This was elaborated by a CHMT member as follows:

“In the past you really only [pointed out] the problems… only problems. There were no congratulations to them [the healthcare provider]. There was no [thing like] telling them that at least they reached some percentages. But with e-TIQH… it shows you ‘Here you did well, here there’s a problem’. …you can see the area in which you have improved, and the area [where] you still have a lot of work. But the old one [routine CHMT supportive supervision] only showed problems. It didn’t show an area where you put efforts in. […] This one [e-TIQH supportive supervision],… it doesn’t discourage you… it shows you the weaknesses and where you did well. So you know it’s possible. At least you are activated [motivated] to continue working.” (CHMT member, Mvomero DC)

Ownership was further increased by the feedback summary form left at the health facility (6/23, observational data). Lastly, although it was intended in the e-TIQH approach to involve the HFGC chair during the feedback at the health facility, we could not find respondents confirming this. However, most interviewees generally supported this idea and saw it as an additional option to further increase feedback acceptance and ownership at health facility level.

#### Reporting

Automated data entry with instant and continuous access to more detailed reports after uploading the surveys ensured timeliness and accessibility of data (18/23). According to respondents this was unlike routine CHMT supportive supervision where data was hardly ever systematically analysed (2/23, observational data), feedbacks delayed (8/23) and reports difficult to access (8/23, observational data). Further benefits of the electronic tool were increased legibility (3/23) and security (3/23) of the data compared to routine CHMT supportive supervision. Importantly, due to the overall improved data quality, the e-TIQH approach also led to more meaningful and actionable data, which could be aggregated and compared at health facility and council level (19/23) as illustrated by a CHSB member:

“We use those [the results of the e-TIQH assessment] because they were being compiled and they show that our facilities had the issues 1, 2, 3. In case of the routine supportive supervision [CHMT] I actually haven’t seen its results [showing that] we visited all facilities [and] we saw that the main problem is this… They produce [results] for individual facilities. But if they were to do it like e-TIQH to compile results [showing] that in all our health facilities it appears as this is the problem… Then this obtains weight during the planning [and] if it’s common [to all facilities], it is necessary to plan for this. (…) Thus, during implementation it gets priority. (…) Thus, it [e-TIQH] gives you an overview of the whole district [council] showing the problem is this, but the other one of the CHMT it was like individual [data]” (CHSB member, Rufiji DC)

In contrast, lower data quality of routine CHMT supportive supervision reduced its usefulness (15/23). Consequently, respondents reported that it was difficult to keep track of what needed to be addressed (8/15), do follow-ups (12/15) and monitor changes (10/15), which led to untimely and/or inadequate actions (8/15) and ultimately to no or only slow improvements. A CHMT and a facility in-charge stated their point of view as follows:

“When coming back [to the facility] for another supervision you may or may not find the [previous supportive supervision] report. Thus, you might not know anymore where the problem was. This is different from now… once uploaded, even at the office you have the file… Thus, it’s easy, even when going back another time you exactly know ‘There I left with this particular problem at that time. Now let me follow up and see how far they’ve come.’”(CHMT member, Mvomero DC)

#### Dissemination

Having access to comparable health facility results (as it was the case during the annual dissemination meeting) contributed to increased result acceptance (18/23), ownership of quality improvement initiatives (16/23) and motivation (20/23) amongst all stakeholders. This was summarized by a facility in-charge:

“In the past this [dissemination meeting] was not done… They [CHMT] came, did supervision and left to do their [work] (…) Completely different from e-TIQH, because when they came [for the dissemination meeting] they transparently displayed for the whole district [council] how we deliver our services and where the weaknesses are […] I used to believe that maybe I was the only one with challenges, but when I arrived there, [I saw] there are colleagues of mine, whose conditions were very bad… So, at least I got motivated [that]… I had to work hard in order to reach another level… I was very pleased because I realized that I already reached a certain position. Thus, [I asked myself] what should I do in order to move further?” (Facility in-charge, Mvomero DC)

The annual dissemination meeting with all relevant stakeholders was seen as a crucial forum for mutual learning and understanding, where best practices, lessons learned, success and failures of quality improvement initiatives could be shared (8/23).

### Cost assessment

Table 4 shows personnel costs (based on salary and time spent) and financial costs (per diems/allowances, transport, and other expenses) of introducing e-TIQH supportive supervision in a new council. The first three activities in Table 4 were part of the e-TIQH supportive supervision approach in the past. The one-day platform usage training was added based on findings from the qualitative study, which pointed out a lack of capacity within the CHMT to fully use the results generated by the e-TIQH assessments. Overall and financial cost was lower than one round of routine supervision in a rural council. In an urban council financial cost was around 1.7 times the financial cost of one round of routine CHMT supervision, leading also to higher overall cost.

**Table 4:** Cost of introducing e-TIQH supportive supervision in a new council in 2016 USD by type of council, resource and activity.

	Rural			Urban		
	*Personnel cost*^4^	*Financial cost*^5^	*Total*	*Personnel cost*^4^	*Financial cost*^5^	*Total*
1 day sensitization meeting^1^	1'361	1’070	2'431	1'190	740	1'930
2 days start-up training^2^	1'439	1'234	2'673	1'439	1’006	2'445
Implementation supervision by 2 trainers	976	448	1'424	767	503	1'270
1 day platform usage training^3^	552	503	1'055	552	471	1'022
***Total***	***4'327***	***3'256***	***7'583***	***3'948***	***2'720***	***6’667***

Figures are rounded and thus might not exactly add up to the total

^1^Participant composition: 5 Council officials, 12 CHMT members, 5 non-CHMT assessors and 2 trainers with one driver

^2^Participant composition: 12 CHMT members, 5 non-CHMT assessors and 2 trainers

^3^Participant composition: 8 CHMT members and 2 trainers

^4^Personnel cost includes the time spent by staff based on their salary

^5^Financial cost includes per diems/allowances, transport for trainers (300km one way from regional headquarter) and other expenses, like supplies (e.g. print outs, notebook), rent, food and refreshment during meeting and trainings

**Fig 6:**
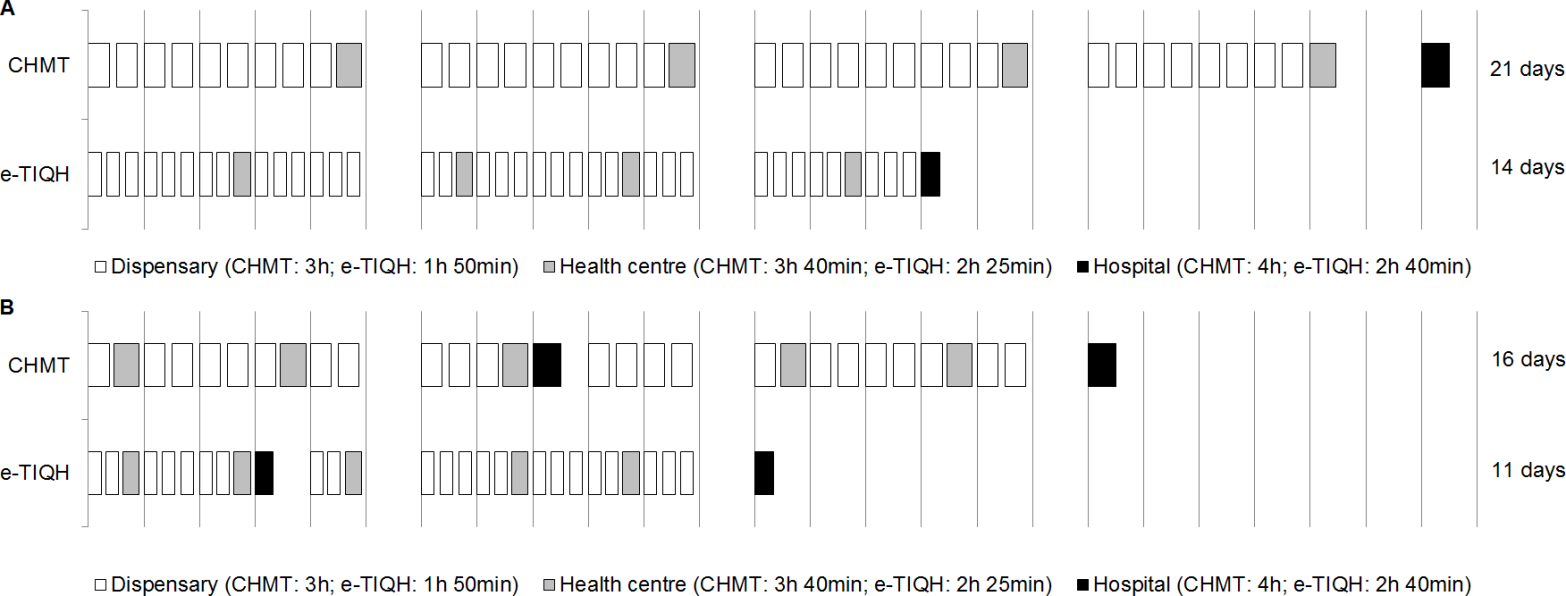
Possible supportive supervision schedule showing assessment days required by the supportive supervision approach in an average rural (A) and urban (B) council. Vertical lines indicate a working day, consisting of eight hours (08:00–16:00). For simplicity schedule presented was developed for one team assessing the whole council.

Table 5 shows hours required by the assessment teams for one round of routine CHMT and e-TIQH supportive supervision by type of council and activity. The biggest task was visiting and assessing all health facilities, which was less during e-TIQH supportive supervision compared to the routine CHMT approach. Importantly, this not only decreased the time required by the assessors, but also the time and burden for the healthcare providers. The time valuation of the latter was not incorporated in the results presented in Table 5. Less time spent at the health facility also allowed assessing more health facilities within one day. This reduced the overall number of days required to visit all health facilities within an average rural and urban council as shown illustratively in Fig 6. Besides the time needed for conducting the assessment, e-TIQH supportive supervision also reduced time spent on reporting.

**Table 5:** Estimated hours required by the assessment team for one round of routine CHMT and e-TIQH supportive supervision, by type of council and activity.

	Routine CHMT supportive supervision		e-TQIH supportive supervision			
			Recommended option		Reduced assessor option	
	*Rural*	*Urban*	*Rural*	*Urban*	*Rural*	*Urban*
Preparation	34	34	41	41	34	34
Implementaion^1^	1’008	768	784	616	672	528
Reporting	147°	134°	116	116	97	97
***Total***	***1189***	***936***	***941***	***773***	***803***	***659***

°Data entry after supportive supervision was assumed to take three minutes per page

^1^Only includes time of the assessors and not time spent by the healthcare provider taking care of the assessment team

Further information about time spent on more specific activities can be found in S2 Table in supporting information

The overall decrease in time used for supportive supervision by the e-TIQH approach also translated into lower personnel and financial (per diems/allowances, transport, and other expenses) costs (Table 6). This was the case in all scenarios presented in Table 6, except for the recommended option in an average urban council, where financial cost was slightly higher despite clearly lower overall cost. The reason for this was the cost of other expenses, which included tablet (16USD/tablet) and platform running (92USD/council) costs. Time spent doing the assessment turned out to be the main cost driver, because of the amounts spent on per diems. Interestingly, overall cost and per diem cost were lower during e-TIQH supportive supervision despite the fact that the assessment team consisted of two more assessors than in the CHMT approach (Table 6A and 6B). If an equal amount of assessors was to be used, the decrease would be even more pronounced (Table 6C). Yet, this is likely to impact acceptance of the assessment amongst stakeholders involved as it would reduce the diversity of the assessment team’s perspectives and therewith affect effectiveness of supportive supervision.

**Table 6:** **Cost for one round of CHMT (A) and e-TIQH supportive supervision (B&C) in 2016 USD by type of council, resource and activity**.

	**Routine CHMT supportive supervision**											
**A**	Rural						Urban					
	*Personnel*	*Per diem/ allowance*	*Transport*^1^	*Otder expenses*^2^	***Total financial***	***Total overall***	*Personnel*	*Per diem/ allowance*	*Transport*^1^	*Otder expenses*^2^	***Total financial***	***Total overall***
Preparation	145	0	0	55	***55***	***199***	143	0	0	43	***43***	***187***
Implementation^4^	3'782	3'479	571	18	***4’069***	***7'851***	2'881	1'325	229	14	***1’568***	***4'449***
Reporting	626	0	0	1	***1***	***627***	573	0	0	1	***1***	***574***
***Total***	**4'553**	***3'479***	***571***	***74***	***4’124***	***8'677***	***3'598***	***1'325***	***229***	***58***	***1’612***	***5'210***
	**e-TIQH supportive supervision—Recommended option**											
**B**	Rural						Urban					
	*Personnel*^3^	*Per diem/ allowance*	*Transport*^1^	*Other expenses*^2^	***Total financial***	***Total overall***	*Personnel*^*3*^	*Per diem/ allowance*	*Transport*^1^	*Other expenses*^2^	***Total financial***	***Total overall***
Preparation	174	146	9	22	***177***	***352***	174	55	5	20	***79***	***253***
Implementation^5^	2'999	2'687	571	215°	***3’474***	***6'473***	2'356	1'056	229	215°	***1’500***	***3'856***
Reporting	496	146	9	2	***157***	***653***	496	55	5	2	***61***	***557***
***Total***	**3'669**	***2'980***	***590***	***240***	***3’809***	***7'478***	***3'026***	***1'165***	***238***	***237***	***1’640***	***4'666***
	**e-TIQH supportive supervision—Reduced assessor option**											
**C**	Rural						Urban					
	*Personne*l^3^	*Per diem/ allowance*	*Transport*^1^	*Other expenses*^2^	***Total financial***	***Total overall***	*Personnel*^*3*^	*Per diem/ allowance*	*Transport*^1^	*Other expenses*^2^	***Total financial***	***Total overall***
Preparation	146	73	5	22	***100***	***246***	146	27	2	20	***50***	***195***
Implementation^6^	2'521	2'303	571	199°	***3’074***	***5'595***	1'981	905	229	199°	***1’333***	***3'314***
Reporting	413	73	5	2	***80***	***493***	413	27	2	2	***31***	***444***
***Total***	***3'080***	***2'450***	***580***	***223***	***3’254***	***6'334***	***2'540***	***960***	***233***	***221***	***1’414***	***3'954***

Figures are rounded and thus might not exactly add up to the total

°Included cost for tablets and the platform running cost assuming the latter would be shared across all 179 councils in Tanzania. Without tablets the figure would be 16USD/tablet lower and without platform running cost 92USD/council.

^1^Included transport allowances

^2^Others expenses included supplies (e.g. print outs, notebook, tablets) as well as communication, internet and platform running cost

^3^Depending on which non-CHMT members will be selected, they might not be on government payroll. However, it was assumed that there personnel cost would be the same as in the case of a CHMT member assessor

^4^Assessment team consists of twice five CHMT members (in total 10 assessor) with one driver each

^5^Assessment team consists of twice four CHMT members and two non-CHMT members (in total 12 assessor) with one driver each

^6^Assessment team consists of twice four CHMT members and one non-CHMT member (in total 10 assessor) with one driver each

Further information about the cost of more specific resources can be found in S1 Table in supporting information

Finally, the cost for conducting an annual dissemination meeting is given in Table 7. Financial cost and therewith overall cost in a rural council for this meeting exceeded the amount of one full round of routine CHMT supportive supervision due to per diem rates paid to participants. In an urban council financial and overall dissemination meeting costs remained lower than one round of supportive supervision. This was due to the proximity of the health facilities to the council headquarter resulting in less expenses for per diems and less time spent travelling to the meeting.

**Table 7:** Annual dissemination meeting cost in 2016 USD by type of council, resource and activity.

	Rural			Urban			
	*Personnel cost*^3^	*Financial cost*^4^	*Total*		*Personnel cost*^3^	*Financial cost*^4^	*Total*
Preparation^1^	136	9	146		136	9	146
1 day dissemination meeting^2^	3'622	6'120	9'743		1'743	1'407	3'149
***Total***	***3'759***	***6'130***	***9'888***		***1'879***	***1'416***	***3'295***

Figures are rounded and thus might not exactly add up to the total

^1^Preperation done by 2 CHMT members during two days

^2^Participant composition: 5 Council officials, 12 CHMT members, 7 CHSB members, 40 (rural) / 30 (urban) health facility in-charge, 32 (rural) / 14 (urban) HFGC chair [42]

^3^Personnel cost includes the time spent by staff based on their salary

^4^Finacial cost includes per diems/allowances, transport and other expenses like supplies (e.g. print outs, notebook), communication cost as well as rent, food and refreshment during meeting and trainings

## Discussion

Findings with regard to routine CHMT supportive supervision were well in-line with what has been reported previously for Tanzania or similar settings [4, 5, 10, 12, 15, 20, 24–29, 31–33]. Our results also revealed the advantages and challenges of e-TIQH supportive supervision, as well as issues of routine CHMT supportive supervision that still remain with the e-TIQH approach.

### Advantages of e-TIQH supportive supervision

The e-TIQH supportive supervision approach addressed several frequently mentioned challenges of routine CHMT supportive supervision and thus received substantial support at council and health facility level [10, 20, 24–29, 31–33].

#### Financial and human resources

Both qualitative findings and costing results demonstrated that e-TIQH supportive supervision reduced time and cost spent, despite a higher number of assessors needed per team. This allowed saving precious time of overburdened council and health facility staff as well as reducing the need for financial resources. Additionally, owing to the mixed assessment team in the e-TIQH approach only four and not five CHMT members per team were required, thereby further reducing the staff demands on the CHMT’s side. Thus, the more efficient use of human and financial resources could make supportive supervision implementation more feasible and therewith more likely to happen.

#### Data quality

Importantly, our findings showed that e-TIQH supportive supervision also improved availability of evidence through the better quality of collected data. The electronic format of the tool in particular increased completeness, legibility, timeliness, accessibility, security and meaningfulness of the data, as well as the user-friendliness of the assessment. Automated data entry and analysis facilitated simple and immediate access to aggregated and comparable data and eliminated the problem of manual data entry errors. This was found to be a major improvement compared to routine CHMT supportive supervision, where data entry and systematic analysis was hardly ever done.

Other features of the e-TIQH approach also contributed to improved data quality. For example the improved assessment design, in particular the multi-dimensional quality concept and the assessment’s focus on processes and structural adequacy, increased perceived accuracy and acceptance of the assessment [38]. This was the case although the e-TIQH assessment tool did not have a higher number of indicators. In fact, the clearly defined and more concise indicator set improved reliability of the assessment. Thus, fewer, but more accurate indicators that are consistently followed up might lead to more substantial improvements than a more comprehensive indicator set, which is not consistently followed-up [38].

Also, a more diverse assessment team, involving CHMT core and co-opted members as well as community and private sector representatives, reduced bias and further increased perceived accuracy and acceptance of the assessment. Interestingly, the national supportive supervision guidelines already stipulate the need of such mixed teams, but it has not been implemented so far [20, 31, 33].

#### Feedback at health facility

The way comprehensive and action-oriented feedback was given to all stakeholders at health facility level was another key feature of the e-TIQH approach. Instead of primarily focusing on negative aspects, the language was supportive and the advices were constructive and summarized in writing. Also, joint discussions were solution-oriented and clear, achievable tasks were assigned to all stakeholders involved. Thus, as hypothesised by Mboya et al. [37], the feedback led to increased knowledge and skills, was more accepted and improved motivation and ownership of subsequent quality improvement measures at facility level [39].The need for constructive and supportive feedback is supported by other literature showing its importance for effective supportive supervision [4, 5, 24, 26, 48–52].

#### Data usage

Overall, the improved data quality of the e-TIQH approach allowed for more systematic follow ups, better monitoring of changes as well as more timely and adequate actions. Additionally, as speculated by Mboya et al. [37], the approach increased usage of collected data during planning and budgeting, leading to more evidence-based resource allocation. If used at national scale the e-TIQH supportive supervision approach would also allow for comparison between councils and regions, addressing another major gap of current routine CHMT supportive supervision [20]. This further opens up the opportunity to strengthen and facilitate the role of RHMTs in supervising CHMTs, which in turn would be likely to stimulate motivation and ownership of CHMTs to conduct adequate supportive supervision. At the same time, the e-TIQH approach would offer a great possibility to overcome the lack of national indicators for monitoring quality of healthcare and ensure improved alignment with indicators of vertical programs, development partners and national accreditation initiatives [18, 20, 27, 28, 31, 53]. The later was for example the case in Rufiji DC, a council where a national star rating system was introduced in 2016 (Table 1) [53]. Most importantly, by making the e-TIQH approach the standard approach for routine CHMT supportive supervision, it would ensure that required improvements are actually happening and therewith would accelerate the ministry’s efforts to move towards accreditation of all health facilities [31].

### Challenges to the e-TIQH supportive supervision

#### Financial resources

Financial concerns in terms of purchasing tablets and covering platform cost could not be confirmed, as they represented only a small part of the overall cost. Additionally, tablets could be substituted by personal smart phones and an open-source platform could be made available to reduce running cost in the medium to long term. One-time financial start-up cost for introducing e-TIQH supportive supervision in a council was within the range of one round of supervision, depending on the type of council. In contrast, the financial cost for conducting an annual dissemination meeting, which exceeded the amount of one round of supportive supervision in rural councils, would occur yearly. Thus, it may be difficult to maintain the implementation of dissemination meetings in resource constraint settings. However, qualitative data indicated that dissemination meetings contributed substantially to increased knowledge and skills through mutual learning and understanding. It thereby supported the aim of the government to promote peer learning and exchange of experiences [18]. The dissemination meetings also strongly improved result acceptance, ownership of quality improvement measures and motivation amongst all stakeholders, similar to what has been shown elsewhere [54]. Thus, it was effective in rewarding good health facility performance without financial incentives. Spotlighting quality of care set the bar for performance and managed to create a system for recognition, something which is well known to improve motivation and retention of healthcare providers [3, 33, 55–57]. This was the case despite the fact that two out of three study councils already had experiences with pay-for-performance (P4P) schemes (Table 1).

### Remaining challenges for supportive supervision

The results presented here also revealed issues of routine CHMT supportive supervision that e-TIQH supportive supervision could not overcome. One of the main remaining challenges is the competing tasks and ad-hoc assignments among CHMT members, leading to the disruption of planned supportive supervision, which was similar to findings from previous studies [25, 26, 31, 32]. Additionally, insufficient and delayed financial resources and availability of vehicles for supportive supervision would remain a major challenge and affect motivation of CHMT members, in-line with what has been raised by others [25, 26, 29, 30]. Neither will e-TIQH nor routine CHMT supportive supervision be effectively implemented with insufficient assessors or assessors lacking contextual knowledge or professional and organisational skills [25].

### Limitations of the study

Although findings presented here were supported by triangulation of methods, causality between the e-TIQH approach and objectively measured improvements in supportive supervision cannot conclusively be claimed. Especially, it remains uncertain how much of the improvement was attributable to the usage of an electronic tool, and how much was due to the overall e-TIQH approach and spirit. Also, it could not be excluded that the ISAQH staff influenced the results presented here. In particular during the dissemination they played a major role as they were the ones presenting the results. For the health facility assessment and subsequent feedback the ISAQH staff only acted as facilitators, while the CHMT members were conducting the activities. Additionally, it is likely that the organisational capacity of the ISAQH staff was greater than the one of an average CHMT, which might have smoothened implementation of the supportive supervision exercise. This also means it is not clear if without the ISAQH staff the e-TIQH approach would be rolled out in routine practices as reasonable. Similar considerations apply for the financial resources for implementation that were readily accessible through project funds and might have influenced CHMT member motivation, especially because per diems were paid in time. Thus, it remains unclear to which extent implementation will be successful in the absence of some form of project support.

The economic costing relied on reported estimates of time used by a small sample of CHMT members. These estimates could not be validated to ensure reported time would reflect actual time spent. Also, sense of time was likely to have varied between respondents. However, to address this, only estimations from CHMT members who participated in both approaches were considered in our study. It should be further recognized that there might have been some recall bias as the interviews took place one to two years after the last implementation of the e-TIQH approach.

It has also to be acknowledged that the respondents were aware of the link between interviewers and the team facilitating the implementation of the e-TIQH supportive supervision approach. This could have potentially led to statements overestimating the contribution of the e-TIQH approach.

Finally, none of the studies aimed to examine the effects of the e-TIQH-linked quality improvements on changes in health outcomes. Hence, the proof that improved processes lead to improved outcomes should be the subject of further research, for example through linking community health data with health facility data.

## Conclusion

Compared to routine CHMT supportive supervision, the e-TIQH supportive supervision approach increased healthcare providers’ knowledge and skills, as well as the quality of data collected and acceptance of supportive supervision amongst stakeholders involved. It additionally ensured better availability of evidence for follow-up actions, including budgeting and planning, and higher stakeholder motivation and ownership of subsequent quality improvement measures. Therewith it facilitated achieving and maintaining crucial quality standards, which ultimately lead to improvements in quality of primary healthcare [39]. The e-TIQH supportive supervision approach also reduced time and cost spent during supportive supervision. This increased feasibility of supportive supervision and hence the likelihood of its implementation. Thus, the results presented together with previous findings suggested that if used as the standard approach for routine CHMT supportive supervision the e-TIQH approach offers a suitable option to make supportive supervision more efficient and effective and therewith more sustainable [37–39]. The e-TIQH approach not only addressed specific challenges frequently experienced with routine CHMT supportive supervision in Tanzania but also provides informed guidance to overcome several problems of supportive supervision and healthcare quality assessments in low- and middle income countries [3, 5, 15, 58–60]. Therefore, it may prove useful for enhancing quality of care in such settings.

## Supporting information

S1 Table Unit cost of resources in Tanzanian Shillings (TSh). Exchange rate in 2016 was 2’188TSh per USD. ^1^Source: Assumption based on information given by respondents and national salary scales [46]; ^2^Source: Personal communication; ^3^Salary and location-dependent; source: Information given by respondents, cross verified by official documentation collected by SR and IM; ^4^Said to be half of the lowest per diem rate (village level); source: information and assumptions given by respondents, cross verified by personal communication; ^5^Source: Information given by respondent, cross verified by CCHP budgets and quarterly combined TFPIRs collected by SR and IM; ^6^Source: CCHP budgets collected by SR and IM; ^7^Source: Market price collected by SR and IM; ^8^Source: ISAQH documents collected by SR and IM.
(DOCX)

S2 Table Estimated quantity and time required for CHMT and e-TIQH supportive supervision by activity (average across all three study councils). (DOCX)

## Abbreviations

CCHP: Comprehensive Council Health Plan
CHMT: Council Health Management Team
CHSB: Council Health Service Board
DC: District Council
e-TIQH: lectronic Tool to Improve Quality of Healthcare
HFGC: Health Facility Governing Committee
IMC: Integrated Management Cascade
ISAQH: Initiative to Strengthen Affordability and Quality of Healthcare
MC: Municipal Council
NIMR: National Institute for Medical Research
P4P: Pay-for-Performance
RHMT: Regional Health Management Team
TFPIR: Technical and Financial Performance Implementation Report
TSh: Tanzanian Shilling
