# Supplementary material for: Towards improved health service quality in Tanzania: An approach to increase efficiency and effectiveness of routine supportive supervision
Source: PLoS One. 2018 Sep 7;13(9):e0202735. doi: 10.1371/journal.pone.0202735 (PMC6128487; doi:10.1371/journal.pone.0202735)
Supplement: S1 Table — Exchange rate in 2016 was 2’188TSh per USD. 1Source: Assumption based on information given by respondents and national salary scales [46]; 2Source: Personal communication; 3Salary and location-dependent; source: Information given by respondents, cross verified by official documentation collected by SR and IM; 4Said to be half of the lowest per diem rate (village level); source: information and assumptions given by respondents, cross verified by personal communication; 5Source: Information given by respondent, cross verified by CCHP budgets and quarterly combined TFPIRs collected by SR and IM; 6Source: CCHP budgets collected by SR and IM; 7Source: Market price collected by SR and IM; 8Source: ISAQH documents collected by SR and IM. (DOCX) [file pone.0202735.s001.docx]

**Table S1** Unit cost of resources in Tanzanian Shillings (TSh).

| **Item** | **Unit cost** |
| --- | --- |
| Average salary of trainers^1^ | 19’067/h° |
| Average salary of council officials^1^ | 18’115/h° |
| Average salary of CHMT/non-CHMT assessor/CHSB^1^ | 9'332/h° |
| Average salary of health facility in-charge (health centre)^1^ | 5’331/h° |
| Average salary of health facility in-charge (dispensary)^1^ | 5’122/h° |
| Average salary of HFGC chair^1^ | 3’842/h° |
| Average salary of driver^2^ | 2'596/h° |
| Per diem trainer (village level)^3^ | 70’000/day |
| Per diem trainer (council level)^3^ | 100’000/day |
| Per diem CHMT (village level)^3^ | 62'500/day |
| Per diem non-CHMT assessor (village level)^3^ | 60’000/day |
| Per diem non-CHMT assessor/CHSB (council level) ^3^ | 80'000/day |
| Per diem health facility in-charge (health centre) (council level)^3^ | 80’000/day |
| Per diem health facility in-charge (dispensary) (council level)^3^ | 73’333/day |
| Per diem HFGC chair (council level)^3^ | 60’000/day |
| Per diem driver (village level)^3^ | 50'000/day |
| Per diem driver (council level)^3^ | 60’000/day |
| Extra duty allowance for council officials^4^ | 35’000/day |
| Extra duty allowance CHMT^4^ | 31'250/day |
| Extra duty allowance non-CHMT assessor/CHSB^4^ | 30’000/day |
| Extra duty allowance health facility in-charge (health centre)^4^ | 30’000/day |
| Extra duty allowance health facility in-charge (dispensary)^4^ | 28’333/day |
| Extra duty allowance HFGC chair^4^ | 25’000/day |
| Extra duty allowance driver^4^ | 25'000/day |
| Transport allowance (Rural council)^5^ | 5'000/way |
| Transport allowance (Urban council)^5^ | 2'500/way |
| Diesel (1L per 7km)^6^ | 2'500/L |
| Rent for conference facility^6^ | 100’000/day |
| Food and refreshment per person^6^ | 10’000/day |
| Print out of page^7^ | 50/page |
| Communication voucher^7^ | 10'000/week |
| Notebook^7^ | 700 each |
| Pen^7^ | 300 each |
| Internet bundle^7^ | 8'333/week |
| Multi plug (durable for 3 years=12 rounds)^7^ | 25'000 each |
| Tablet (durable for 3 years=12 rounds)^7^ | 200'000 each |
| Training material^8^ | 3’000 each |
| Annual platform running cost (179 councils with 12 users each)^8^ | 808'124/council |

Exchange rate in 2016 was 2’188TSh per USD

°Yearly salary was assumed to be equal to 52 weeks of 40 hours of work

^1^Source: Assumption based on information given by respondents and national salary scales (47)

^2^Source: Personal communication

^3^Salary and location-dependent; source: Information given by respondents, cross verified by official documentation collected by SR and IM

^4^Said to be half of the lowest per diem rate (village level); source: information and assumptions given by respondents, cross verified by personal communication

^5^Source: Information given by respondent, cross verified by CCHP budgets and quarterly combined TFPIRs collected by SR and IM

^6^Source: CCHP budgets collected by SR and IM

^7^Source: Market price collected by SR and IM

^8^Source: ISAQH documents collected by SR and IM
