# Supplementary material for: Towards improved health service quality in Tanzania: An approach to increase efficiency and effectiveness of routine supportive supervision
Source: PLoS One. 2018 Sep 7;13(9):e0202735. doi: 10.1371/journal.pone.0202735 (PMC6128487; doi:10.1371/journal.pone.0202735)
Supplement: S2 Table — (DOCX) [file pone.0202735.s002.docx]

**Table S2** Estimated quantity and time required for CHMT and e-TIQH supportive supervision by activity (average across all three study councils).

|  | **Routine CHMT supportive supervision** | |  | **e-TIQH supportive supervision** | |
| --- | --- | --- | --- | --- | --- |
|  | *Quantity* | *Time required* |  | *Quantity* | *Time required* |
| ***Preparation*** |  |  |  |  |  |
| Prepare checklist | 2x25pages per health facility | 90sec/40pages |  | ----------------- | ----------------- |
| Update surveys | ----------------- | ----------------- |  | 12 devices | 5min/device |
| Prepare feedback summary form | ----------------- | ----------------- |  | 2x5pages per health facility | 90sec/40pages |
| Preparatory meeting and logistics | 10 CHMT members | 3h13min/person |  | 8 CHMT members and 4 non-CHMT members | 3h13min/person |
| Introduction of a new team member | 2 CHMT members | 15min/person |  | 2 CHMT members | 30min/person |
| ***Implementation*** |  |  |  |  |  |
| Charge devices | ----------------- | ----------------- |  | 1 CHMT member per team and day | 7min/6tablets |
| Introduction at the health facility | 5 CHMT members | 11min/health facility |  | 4 CHMT members and 2 non-CHMT member | 9min/health facility |
| Data collection at the health facility | 5 CHMT members | 1h48min/  dispensary; 2h30min/  health centre; 2h54min/  hospital |  | 4 CHMT members and 2 non-CHMT member | 1h/dispensary; 1h30min/health centre; 1h48min/hospital |
| Production of feedback at the health facility | 5 CHMT members | 29min/health facility |  | 4 CHMT members and 2 non-CHMT member | 18min/health facility |
| Provision of feedback at the health facility | 5 CHMT members | 29min/health facility |  | 4 CHMT members and 2 non-CHMT member | 24min/health facility |
| Data processing | ----------------- | ----------------- |  | 1 CHMT member per team | 1h30min/day |
| 1 round of supportive supervision | 2 teams of 5 CHMT members and 1 driver each | 10.5 days/rural council; 8 days/ urban council |  | 2 teams of 4 CHMT members, 2 non-CHMT member and 1 driver each | 7 days/rural council; 5.5 days/ urban council |
| ***Reporting*** |  |  |  |  |  |
| Data entry | 1 CHMT member for each team | 3min/page |  | ----------------- | ----------------- |
| Discussion and report writing (1 page per facility) | 10 CHMT members | 9h41min/person |  | ----------------- | ----------------- |
| Discussion and report writing (2 pages/facility & 10 pages/council) | ----------------- | ----------------- |  | 8 CHMT members and 4 non-CHMT members | 9h41min/person |
